# Electronic Medical Records as Input to Predict Postoperative Immediate Remission of Cushing’s Disease: Application of Word Embedding

**DOI:** 10.3389/fonc.2021.754882

**Published:** 2021-10-13

**Authors:** Wentai Zhang, Dongfang Li, Ming Feng, Baotian Hu, Yanghua Fan, Qingcai Chen, Renzhi Wang

**Affiliations:** ^1^ Department of Neurosurgery, Chinese Academy of Medical Sciences and Peking Union Medical College, Peking Union Medical College Hospital, Beijing, China; ^2^ School of Computer Science, and Technology, Harbin Institute of Technology (Shenzhen), Shenzhen, China; ^3^ Department of Neurosurgery, Beijing Tiantan Hospital, Beijing Neurosurgical Institute, Capital Medical University, Beijing, China; ^4^ Peng Cheng Laboratory, Shenzhen, China

**Keywords:** natural language processing, Cushing’s disease, immediate remission, preoperative prediction, machine learning

## Abstract

**Background:**

No existing machine learning (ML)-based models use free text from electronic medical records (EMR) as input to predict immediate remission (IR) of Cushing’s disease (CD) after transsphenoidal surgery.

**Purpose:**

The aim of the present study is to develop an ML-based model that uses EMR that include both structured features and free text as input to preoperatively predict IR after transsphenoidal surgery.

**Methods:**

A total of 419 patients with CD from Peking Union Medical College Hospital were enrolled between January 2014 and August 2020. The EMR of the patients were embedded and transformed into low-dimensional dense vectors that can be included in four ML-based models together with structured features. The area under the curve (AUC) of receiver operating characteristic curves was used to evaluate the performance of the models.

**Results:**

The overall remission rate of the 419 patients was 75.7%. From the results of logistic multivariate analysis, operation (*p* < 0.001), invasion of cavernous sinus from MRI (*p* = 0.046), and ACTH (*p* = 0.024) were strongly correlated with IR. The AUC values for the four ML-based models ranged from 0.686 to 0.793. The highest AUC value (0.793) was for logistic regression when 11 structured features and “individual conclusions of the case by doctor” were included.

**Conclusion:**

An ML-based model was developed using both structured and unstructured features (after being processed using a word embedding method) as input to preoperatively predict postoperative IR.

## Introduction

Pituitary corticotroph adenoma is also called Cushing’s disease (CD). It accounts for the majority of Cushing’s syndrome cases (1, 2). Cushing’s syndrome causes various types of symptoms and signs, such as central obesity, supraclavicular fat accumulation, thinned skin, purple striae, proximal muscle weakness, fatigue, high blood pressure, glucose intolerance, acne, hirsutism, and neurological deficits (3). The first-line treatment method is transsphenoidal surgery (TSS) according to a consensus statement (4). Thus, immediate remission (IR) is important for both patients and surgeons. A previous systemic review showed that the overall IR rate was 77% (52.1%–96.6%) (5).

Several studies have been conducted to investigate perioperative risk factors for the prediction of postoperative prognosis using both traditional biostatistical and machine learning (ML) methods (6–9). ML is a computer-based method for data analysis based on the theory that there are patterns hidden in data, and it helps to predict the prognosis of diseases (10). ML enables a computer to construct models by iteratively learning from the patterns in the dataset. Therefore, an ML-based model is formed based on learning from real-world data rather than learning from doctors’ experience, which may be limited (11). In recent years, there have been an increasing number of ML-related studies on pituitary adenoma. For example, Liu et al. used seven ML-based models that incorporated 17 clinical variables to preoperatively predict the recurrence of CD. The model that performed the best was random forest (RF) with an AUC value of 0.781 (8). Fan et al. used six ML-based models that incorporated 12 clinical variables to predict the TSS response. The final model with the highest AUC value of 0.8555 was the GBDT model.

Features including the preoperative and postoperative serum adrenocorticotropic hormone (ACTH) level, postoperative serum cortisol level, age, and preoperative cavernous sinus invasion on MRI (IOMRI) have been shown to be related to postoperative prognosis (8). All risk factors initially considered in previous studies were selected by clinicians according to their clinical experience and related literature. No existing models use electronic medical records (EMR) as input to predict postoperative IR of patients with CD, even though they may contain a great deal of information that is useful for the prediction of IR. In the present study, EMR is included in the model for the preoperative prediction of postoperative IR of CD.

In recent years, EMR has facilitated data accessibility. There are different types of manifestations in patients with CD because of hypercortisolism that may contain information related to the severity of CD. However, the analysis of diverse and massive EMR data remains challenging because of the complex nature of clinical language and the interpretation process. To address these challenges, in this study, natural language processing techniques are used, specifically contextualized word embeddings, to help humans to access this information in free text to improve predictions. Word embedding is a typical type of natural language processing technique, and it is a suitable method for vectorizing free text so that it can be processed by downstream learning models. Although there has been exponential growth in the number of studies involving radiomics methods, the application of word embedding techniques is still limited (12, 13). In those studies, the text part of EMR was incorporated into the ML model as input, which increased the modal and made the input data closer to real-world data (14, 15).

Postoperative IR is important for clinician–patient communication, and it may influence the treatment strategy. The objective of the present study is to develop an ML model to preoperatively predict postoperative IR using both free text from EMR (after being processed by a word embedding technique) and structured features as input.

## Materials and Methods

### Study Population

The present study was approved by the ethical review committee of Peking Union Medical College Hospital (PUMCH). A total of 419 patients with CD were enrolled between January 2014 and August 2020. All surgery was performed by MF.

The inclusion criteria were as follows: (1) manifestations of Cushing’s syndrome; (2) positive result on MRI or negative result on MRI, but CD was strongly suspected according to manifestations; (3) ruling out the possibility of ectopic ACTH syndrome; and (4) plasma cortisol level (8:00 a.m.) > 22.3 μg/dl or 24-h UFC level > 103.5 μg.

### Diagnosis of Cushing’s Disease

All patients had T1-weighted, T2-weighted, and T1-weighted gadolinium-enhanced MRI. Patients whose T1-weighted gadolinium-enhanced MRI showed the negative result of a pituitary tumor had T1-weighted dynamic gadolinium-enhanced MRI. A hypointense region in T1-weighted MRI within the pituitary gland indicated the positive result of a pituitary adenoma. In cases in which the profiles of the potential tumors were inconspicuous, T1-weighted gadolinium-enhanced MRI was required to outline the tumor. A microadenoma was defined as a tumor whose largest diameter was less than 10 mm and a macroadenoma was defined as a tumor whose largest diameter was ≥10 mm. A total of 392/419 participants had histological confirmation of CD, and the diagnosis of CD was based on synthesized evidence that included MRI results, clinical manifestations, results of the low-dose dexamethasone suppression test (LDDST) and high-dose dexamethasone suppression test (HDDST), and pathological results.

All patients underwent a routine combined LDDST and HDDST to verify hypercortisolism and the location of the tumor. In the LDDST, 0.5 mg of dexamethasone was given to the patient every 6 h for 2 days. The LDDST was considered to be suppressed if 24-h UFC was lower than 12.3 μg/24 h on the second day or plasma cortisol was lower than 1.8 μg/dl in the morning of the third day. In the HDDST, 2 mg of dexamethasone was given to the patient every 6 h for 2 days. HDDST was considered to be suppressed if 24-h UFC on the second day or plasma cortisol in the morning of the third day was >50% lower than the original level. The failure of suppression of the LDDST together with successful suppression of the HDDST indicated CD.

In cases in which there was no evidence of a tumor in preoperative MRI, bilateral inferior petrosal sinus sampling with a desmopressin stimulation test was implemented to confirm the location of the tumor. During the desmopressin test process, 10 mg of desmopressin was given to the patient to stimulate the secretion of ACTH. A ratio of ACTH concentration in the inferior petrosal sinus to peripheral concentration that was larger than 2 in the basal state or larger than 3 after desmopressin stimulation indicated a diagnosis of CD.

The diagnosis of CD was based on the combination of compositive evidence, including MRI results, clinical manifestations, results of LDDST and HDDST, and pathological results.

All TSS was performed by one experienced surgeon (MF). The details of the TSS were discussed previously (16). No medical therapy was administered to patients because of a lack of medicine in China.

The resected tissues were examined for pathology and immunohistochemical analysis for ACTH, growth hormone, thyroid-stimulating hormone, luteinizing hormone, follicle-stimulating hormone, prolactin, Ki-67, and P-53.

### Postoperative Management and Immediate Remission

In the first 3 days after TSS (7 days if IR was not achieved), the plasma cortisol level was tested each day. If the cortisol level was lower than 5 μg/dl, glucocorticoid replacement therapy was started. Glucocorticoid replacement therapy started with 100 mg of hydrocortisone twice a day for 3 days following 30 mg of hydrocortisone orally once a day. After being discharged from hospital, patients decreased the dose by 2.5 mg per week until it reached 2.5–5 mg per day. The cessation of the drug was decided by clinicians according to the evaluation of the pituitary function.

IR was defined as a plasma cortisol level (8:00 a.m.) lower than 5 μg/dl or 24-h UFC lower than 20 μg/24 h within 7 days after surgery (17).

### Study Design

The data included 11 structured clinical features and 10 unstructured features. Missing values were replaced by average values. The structured data included gender, age, first operation or not, largest tumor diameter, invasion of cavernous sinus on MRI (IOMRI), sellar floor changes (SFC), disease duration, BMI, 24-h UFC, plasma cortisol (8:00 a.m.), and plasma ACTH (8:00 a.m.). The unstructured data included the chief complaint, history of present illness (HPI), past medical history, record of first ward round by superior surgeon, cautions, transferred-out record (from the endocrinology department), transferred-in record (to the neurosurgery department), characteristics of the case, discussions about cases, and individual conclusions of the case by doctor. The 10 unstructured features were routine features of EMR in PUMCH. “Characteristics of the case” were the records of the unique characteristics of an individual patient. “Discussions about cases” were the meeting summaries about all patients’ conditions by all surgeons in the neurosurgery department of PUMCH. “Individual conclusions of the case by doctor” were the records of the summary of patients’ characteristics provided by MF. “Cautions” were the main points that needed to be noticed about treating patients provided by MF. Transferred-out records were the main points that needed to be noticed about treating patients and basic conditions of the patient provided by the endocrinologist. Transferred-in records were the main points that needed to be noticed about treating patients and basic conditions of the patient provided by the neurosurgeon. The EMR of unstructured features was vectorized using a word embedding method, and could then can be analyzed in a similar manner to structured features.

The *F*-test was used to rank the structured data. The 10 structured features were sequentially included into each model. Then, each model outputs AUC values for different numbers of features. The min–max normalization method was used on the data. The highest AUC values of the four algorithms were used as their baseline values. Ten features of the unstructured data were introduced into each model individually, and the importance of each unstructured feature was ranked according to the change of AUC.

### ML Algorithms

Four ML algorithms were applied: support vector machine (SVM), logistic regression (LR), RF, and multilayer perceptron (MLP). In each ML algorithm, structured data were sequentially introduced into the algorithm according to their rank in the training dataset. Then, in the test dataset, the same process was conducted. In both the training and test datasets, 10-fold cross-validation was performed. Then, a grid search was used to select the best hyperparameters, as discussed elsewhere (7).

### Statistical Analysis

Statistical analysis was performed using RStudio software (1.2.5042), IBM SPSS Statistics 23 (IBM Corporation), and Python. The Shapiro–Wilk test was used to evaluate the normality of continuous variables. Normally distributed variables were displayed as mean ± standard deviation. Non-normally distributed variables were displayed as the interquartile range. The Wilcoxon test was used to compare non-normal distributed continuous variables in the training dataset and test dataset. Categorical variables were analyzed using a chi-squared test or Fisher’s exact test.

### Occlusion Tests

“Occlusion tests” were performed to determine the contributions that the symptomatic entities made to the ML-based models. In the “occlusion tests”, CMeKG (http://cmekg.pcl.ac.cn/) was used to select and delete the symptomatic entities to build a new HPI without symptomatic descriptions of CD. Then, the two HPIs were vectorized and merged into LR together with the structured features. The result demonstrated that the model with the input of the original HPI was conspicuously superior to that with the input of the newly built HPI.

## Results

### Patients’ Characteristics

A total of 419 patients were included in the study between January 2014 and August 2020. Eleven traditionally used predictors were selected in the study: age, gender, first operation (or not), SFC, IOMRI, tumor diameter (microadenoma or macroadenoma), disease duration, BMI, 24-h UFC, morning plasma cortisol level, and morning plasma ACTH level. All the predictors are presented in **Table 1**. The characteristics of the remission and non-remission groups are presented in **Table 2**. From the results of logistic univariate analysis, the first operation (*p* < 0.001), IOMRI (*p* = 0.010), SFC (*p* = 0.011), and ACTH (*p* = 0.009) were strongly correlated with IR. From the results of logistic univariate analysis, the first operation (*p* < 0.001), IOMRI (*p* = 0.046), and ACTH (*p* = 0.024) were strongly correlated with IR (**Table 3**).

**Table 1:** Participants’ characteristics in trainning and test datasets.

Characteristic	Total	Training dataset	Test dataset	*p*-value
**Gender**	419	335	84	0.991
Male	80 (19.09)	64 (19.10)	16 (19.05)	
Female	339 (80.91)	271 (80.90)	68 (80.95)	
**Age (years)**	37.86 ± 13.06	37.94 ± 13.04	37.55 ± 13.06	0.808
**First Operation**				0.492
Yes	359 (85.68)	289 (86.27)	70 (83.33)	
No	60 (14.32)	46 (13.73)	14 (16.67)	
**Diameter**				0.684
Macroadenoma	40 (9.55)	31 (9.25)	9 (10.71)	
Microadenoma	379 (90.45)	304 (90.75)	75 (89.29)	
**IOMRI**				0.098
Invasion	26 (6.21)	19 (5.67)	9 (10.71)	
Non-invasion	393 (93.79)	316 (94.33)	75 (89.29)	
**Sellar Floor**				0.700
Infiltrated	45 (10.74)	35 (10.45)	10 (11.90)	
Normal	374 (89.26)	300 (89.55)	74 (88.10)	
**Disease Duration (months)**	36 (18–72)	36 (18–72)	43.5 (24–84)	0.121
**BMI**	26.14 (24.04–28.93)	26.15 (24.12–29.03)	26.13 (23.51–28.21)	0.476
**24-h UFC (μg)**	426.40 (268.65–716.40)	441.80 (270.63–745.66)	411.68 (243.58–586.41)	0.139
**Cortisol (μg/dl)**	27.15 (22.39–33.01)	27.02 (22.48–32.49)	27.89 (21.59–33.67)	0.715
**ACTH (pg/ml)**	72.6 (49.6–105)	72.9 (50.7–106.5)	66.3 (44.23–95.75)	0.446

**Table 2:** Patients’ characteristics in remission and non-remission groups.

Characteristic	Remission	Non-remission	*p*
**Gender**	317	102	0.195
Male	65 (20.50)	15 (14.71)	
Female	252 (79.50)	87 (85.29)	
**Age (years)**	37.74 ± 13.01	38.25 ± 13.25	0.730
**First Operation**			**0.005**
Yes	287 (90.54)	72 (70.59)	
No	30 (9.46)	30 (29.41)	
**Diameter**			0.206
Macroadenoma	27 (8.52)	13 (12.75)	
Microadenoma	290 (91.48)	89 (87.25)	
**IOMRI**			**0.007**
Invasion	14 (4.42)	12 (11.76)	
Non-invasion	303 (95.58)	90 (88.24)	
**Sellar Floor Changes**			**0.010**
Infiltrated	27 (8.52)	18 (17.65)	
Normal	290 (91.48)	84 (82.35)	
**Disease Duration (months)**	36 (18–72)	48 (20.25–84)	0.256
**BMI**	26 (24.03–28.87)	26.56 (24.30–29.27)	0.448
**24-h UFC (μg)**	412.56 (266.7–675.24)	452.22 (283.92–821.78)	0.337
**Cortisol (μg/dl)**	27.5 (22.03–32.61)	26.72 (23.03–33.87)	0.954
**ACTH (pg/ml)**	68.3 (45.3–104)	86.45 (55.40–114.50)	**0.004**

Bold values in this table represent statistical significance (P < 0.05).

**Table 3:** Logistic univariate and multivariate analysis of the relationship between risk factors and IR.

Characteristics	Univariate analysis			Multivariate analysis		
	OR	95% CI	*p*-value	OR	95% CI	*p*-value
**Gender**	1.496	0.811–2.759	0.197			
**Age (years)**	0.997	0.980–1.014	0.726			
**First Operation**	3.986	2.258–7.036	**<0.001**	3.641	1.996–6.641	**<0.001**
**Diameter**	0.637	0.316–1.687	0.209			
**IOMRI**	0.347	0.155–0.776	**0.010**	0.413	0.17.–0.985	**0.046**
**Sellar Floor Changes**	0.434	0.228–0.827	**0.011**	0.818	0.393–1.703	0.591
**Disease Duration (months)**	0.998	0.994–1.002	0.436			
**BMI**	0.970	0.923–1.021	0.246			
**24-h UFC (μg)**	1.000	1.000–1.000	0.166			
**Cortisol (μg/dl)**	1.001	0.980–1.023	0.898			
**ACTH (pg/ml)**	0.994	0.990–0.999	**0.009**	0.995	0.991–0.999	**0.024**

Bold values in this table represent statistical significance (P < 0.05).

### Predictive Performance of Models

**Figure 1:**
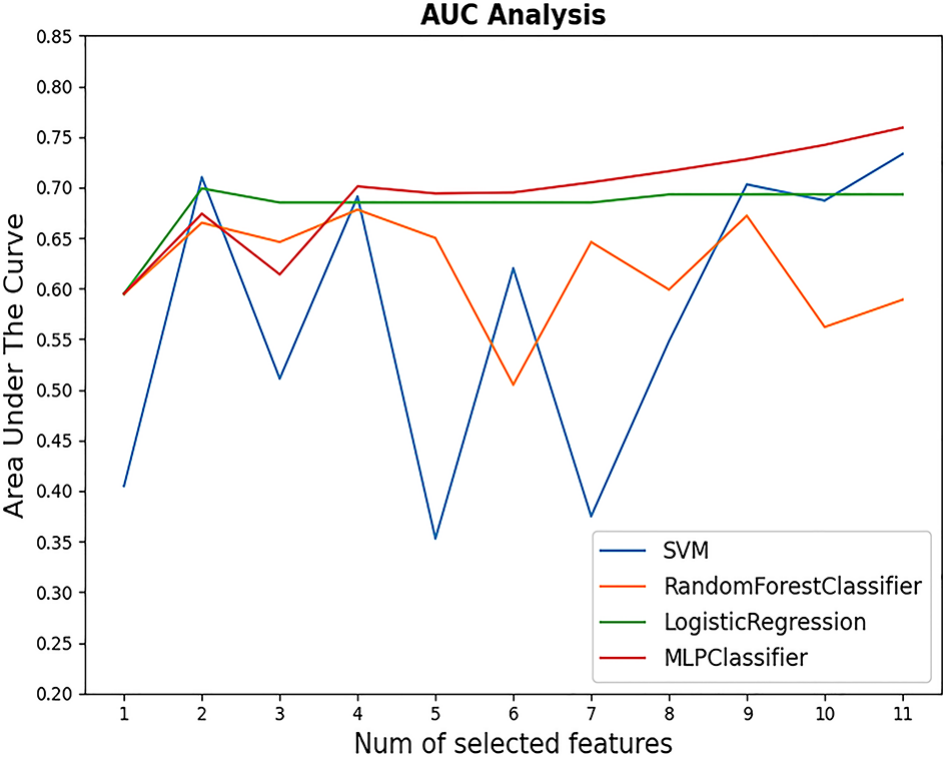
AUC values of four models with different numbers of structured features selected. The highest AUC value appeared when MLP with 11 variables came into use (AUC = 0.759).

**Figure 2:**
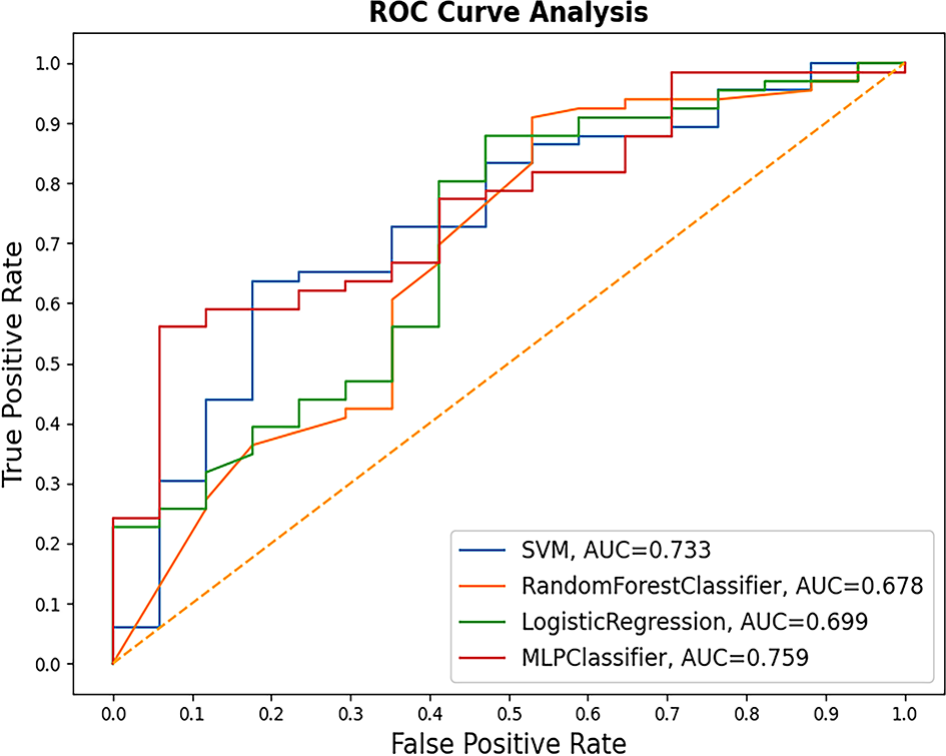
Performances of models with optimal number of structured features. MLP performed the best.

Four ML-based algorithms were used: MLP, SVM, RF, and LR. The performance of each model with different numbers of structured features is shown in **Figure 1**. The highest AUC values for MLP, SVM, RF, and LR were 0.759, 0.733, 0.678, and 0.699, respectively (**Figure 2**). Each unstructured feature was sequentially introduced into the model, which had all structured features included. Then, each model outputs an AUC value (**Table 4**). The chief complaint and individual conclusions of the case by doctor, HPI and individual conclusions of the case by doctor, together with chief complaint and HPI were then introduced into each model; however, the AUC values were not higher than when only one unstructured feature was introduced into the model. The highest AUC value (0.793) was achieved by LR when 11 structured features and individual conclusions of the case by doctor were introduced.

**Table 4:** AUC values and 95 confidence interval of different models with different features.

	MLP	SVM	RF	LR
**Structured data**	**0.759** **[0.633, 0.885]**	**0.733** **[0.612, 0.845]**	0.678 [0.544,0.812]	0.699 [0.594,0.803]
**Chief complaint**	0.729 [0.606, 0.852]	0.661 [0.583,0.739]	**0.686** **[0.610,0.756]**	0.777 [0.709, 0.845]
**HPI**	0.670 [0.552, 0.788]	0.652 [0.570, 0.734]	0.642 [0.549, 0.735]	0.737 [0.624, 0.850]
**Past medical history**	0.606 [0.515, 0.697]	0.610 [0.515, 0.705]	0.556 [0.491,0.621]	0.577 [0.474,0.640]
**RFWR**	0.506 [0.413,0.599]	0.692 [0.572, 0.811]	0.533 [0.425,0.641]	0.573 [0.455,0.690]
**Cautions**	0.619 [0.494, 0.744]	0.718 [0.608, 0.830]	0.468 [0.317,0.619]	0.674 [0.530, 0.818]
**Transferred-out record**	0.473 [0.334,0.611]	0.614 [0.494, 0.734]	0.613 [0.503, 0.723]	0.556 [0.421,0.691]
**Transferred-in record**	0.656 [0.521, 0.791]	0.625 [0.492, 0.758]	0.602 [0.479, 0.725]	0.679 [0.562, 0.796]
**Characteristics of the case**	0.571 [0.484, 0.657]	0.575 [0.432,0.717]	0.474 [0.317,0.630]	0.676 [0.566,0.786]
**Discussions about cases**	0.582 [0.445,0.718]	0.628 [0.556, 0.696]	0.505 [0.430, 0.580]	0.622 [0.505, 0.739]
**Individual conclusions of the case by doctor**	0.723 [0.622, 0.824]	0.682 [0.584,0.780]	**0.686** **[0.623,0.743]**	**0.793** **[0.689, 0.897]**
**Chief complaint and individual conclusions of the case by doctor**	0.669 [0.585,0.753]	0.722 [0.628, 0.816]	0.442 [0.252, 0.632]	0.678 [0.582,0.744]
**HPI and individual conclusions of the case by doctor**	0.737 [0.677, 0.796]	0.691 [0.596, 0.785]	0.499 [0.359, 0.639]	0.678 [0.603,0.753]
**Chief complaint and HPI**	0.680 [0.589,0.771]	0.669 [0.752,0.766]	0.429 [0.295,0.073]	0.721 [0.635, 0.806]

Bold values in this table represent highest AUC value in each ML based model.

Unstructured features contain too much redundant information; hence, three or more unstructured features were not combined in this study to extract valid information.

### Variable Importance

*F*-test univariate analysis was used to rank the importance of the 11 variables. Their rank was as follows: first operation, SFC, morning ACTH, IOMRI, 24-h UFC, disease duration, BMI, tumor diameter, gender, plasma cortisol, and age. The rank of the features of unstructured data was evaluated using the change in AUC value after adding a single unstructured feature into the model based only on the structured features. For LR, “individual conclusions of the case by doctor” was ranked first.

### Occlusion Tests

The performance of the model with the input of the original HPI was conspicuously better than that with the input of the HPI without symptomatic entities (**Table 5**). The red Chinese characters indicate the deleted symptomatic entities.

**Table 5:** Example of Occlusion Test Results.

	Original HPI	HPI after Deletion of Symptomatic Entities by CMeKG
**Symptoms**	患者诉于2年前无明显诱因出现双下肢水肿，乏力，无皮肤菲薄、紫纹。 (The patient complained that edema and weakness in lower limbs appeared 2 years ago without obvious causes. There was no thin skin or purple striae.)	患者诉于2年前无明显诱因出现, 无皮肤菲薄、紫纹。 (The patient complained that appeared 2 years ago without obvious causes. There was no thin skin or purple striae.)
**AUC**	0.737	0.629

## Discussion

TSS is the first-line treatment method for CD. IR rates are typically between 59% and 96.6% (18). In the present study, the IR rate was 75.7% (317/419), which is almost the same as the result of 76% from a previous study (19). IR may be a strong predictor of long-term remission (20). IR is also important for doctor–patient communication because patients are always concerned about whether clinical manifestations can be eliminated immediately. Thus, it is of great importance to develop an ML-based model for the preoperative prediction of IR.

Various types of manifestations exist in patients with CD because of hypercortisolism, such as abnormal fat distribution, weight gain, osteoporosis, diabetes mellitus ecchymosis, and hypokalemia. According to our limited experience, the symptoms and signs a patient has are strongly correlated with the patient’s prognosis. Therefore, we speculate that the unstructured data of patients with CD contributes to the ML-based model for the preoperative prediction of IR. The manifestations of patients with CD may be recorded in EMR, which has been ignored by clinicians in quantitative analysis because natural language could not be processed in the past. However, natural language processing techniques can now deal with EMR as the input of ML-based models, which facilitates the full use of multimodal data (structured data and unstructured data in EMR). In the present study, we performed occlusion tests on HPI and the results demonstrated that the performance of the model with the input of the original HPI was better than that with the input of HPI without symptomatic entities. Therefore, we speculated from the occlusion test and our limited experience that symptomatic entities in HPI were strongly related to IR and conducive to the prediction of IR.

In our previous study, we used several ML algorithms to build ML-based models to preoperatively predict IR (7). In that study, we only included structured data in the ML-based model, whereas in the present study, we introduced not only structured data into the models but also unstructured data. Unstructured data may contain information related to the severity of CD in addition to the 11 features of structured data that were summarized by clinicians according to their personal experience. The features included in the final model with the highest AUC (0.743) in our previous study were IOMRI, tumor size, whether it is the first operation, and ACTH level (8:00 a.m.), whereas in the present study, the model with the highest AUC (0.793) was constructed using LR with 11 structured features and “individual conclusions of the case by doctor.” The model performance in the present study was superior to that in the previous study.

The importance of the features of structured data was ranked using the *F*-test, whereas the importance of the features of unstructured data was evaluated using the change in AUC value after adding a single unstructured feature into the model based only on the structured features. Information such as image and voice, itself has the characteristics of vectorization, continuity. Natural language (EMR) is different. It is the expression and abstract summary of objective things. This is the advantage of human thought; however, it restricts computers to the identification of natural language because it lacks a strong correlation between specific sensory information and natural language. In the past, computers could only perform statistical and logical reasoning through the relationship between symbols, which made it difficult to express the continuity of language. In 2013, Mikolov et al. (21) enabled vocabulary to form the deep model input of continuous real number space in the same manner as images and audio, and the learning efficiency of the model was much higher than that of previous models. Thus, we used a word embedding method in the present study to vectorize EMR. “Individual conclusions of the case by doctor” are routine records in EMR at PUMCH. They are the conclusions of clinicians according to the clinical characteristics of patients, and they may reflect the subjective perception of doctors about the severity of the disease. Therefore, we speculated that key information related to the severity of the disease may be hidden in free text and could contribute to the ML-based model.

**Table 4** shows that the AUC values of MLP and SVM did not increase after unstructured features were introduced into the model, whereas, simultaneously, the AUC values increased significantly after “individual conclusions of the case by doctor” was introduced into the model. These two contrasting results are mainly caused by several factors, as we speculated. First, unstructured data text is generally long, with a great deal of useless information, and can easily be overfitted in MLP, which can lead to the decline of AUC values. Similarly, SVM looks for a hyperplane to separate data points, which makes it difficult to determine an appropriate hyperplane to separate them in the case of complex data features. Therefore, the performance of SVM decreases after unstructured data that contain a great deal of redundant information are introduced into the model. The linear model structure of LR enables it to capture quasi-linear characteristics and ignore high-dimensional redundant information; hence, it can capture key information in the unstructured text to obtain a high-grade classification capacity. To summarize, MLP and SVM are more complex than LR, which made the latter even more effective in the present study.

To the best of our knowledge, the present study is the first to use unstructured data from the EMR of patients with CD as the input of ML-based models. In this process, we embedded these unstructured features, and transformed them into relatively low-dimensional dense vectors to facilitate the model construction of ML (22, 23). In previous studies on the ML model construction process, one-hot encoding on discrete characteristics was typically feasible for clinically used binary structured data (e.g., gender). However, features with one-hot encoding may be too high-dimensional and sparse for EMR data, which is not conducive to model training. CD is a type of neuroendocrine tumor that causes not only a mass effect but also various types of endocrine symptoms recorded in EMR that can be fully used by an ML-based model after embedding.

In the present study, the final model with the highest AUC included all structured features; however, according to the *F*-test, four structured features were correlated with IR. Their rank is as follows: first operation or not, SFC, ACTH, and IOMRI. If a patient has already undergone at least one operation, there is a higher chance that the tumor is more invasive and aggressive, which may cause postoperative residual (24). SFC was the second-most important predictor of IR in the present study. If the sellar floor of a patient is infiltrated on preoperative MRI, it is likely that the tumor has higher invasiveness that makes it invade the mucosa and bone in the sellar region. In this circumstance, there is a relatively great possibility of postoperative residual. Preoperative ACTH level was the third-most important predictor of IR, which is consistent with our previous study (9). IOMRI was the fourth-most important predictor, which is also consistent with our previous study (9). An intriguing observation is that tumor size was not a predictor of IR, which is inconsistent with previous studies (9, 19, 25, 26). In our previous study (7), tumor size was strongly correlated with IR when two surgeons performed operations over several decades. However, in the present study, only MF performed the operation. We can speculate from the result that with the evolution of surgical skills and personal experience, tumor size is no longer a major predictor of IR.

## Strengths and Limitations

The present study has two strengths. First, this is the first study that used deep learning techniques to deal with EMR of patients with CD as input of an ML-based model that improved model performance. EMR contains sufficient information about the patient to reflect real-world information. Second, a relatively large CD cohort was considered. There are also two limitations. First, this was a single-center study. Second, the performance of the ML-based model depended on the quality of EMR.

## Conclusions

EMR of patients with CD can be used as input to an ML-based model after being processed to preoperatively predict IR. The model with structured features together with unstructured features conspicuously enhanced the performance of the model compared with the model that used only structured features as input. First operation or not, SFC, ACTH, and IOMRI were the most important predictors of IR of CD.

## Data Availability Statement

The raw data supporting the conclusions of this article will be made available by the authors, without undue reservation.

## Ethics Statement

The studies involving human participants were reviewed and approved by the ethical review committee of Peking Union Medical College Hospital. Written informed consent to participate in this study was provided by the participants’ legal guardian/next of kin.

## Author Contributions

WZ and DL contributed equally to the present study. Each author contributes to the article in data collecting and analysis. RW and QC take final responsibility for this article. All authors contributed to the article and approved the submitted version.

## Funding

This work was supported by the CAMS Innovation Fund for Medical Sciences (CIFMS) (2020-I2M-C&T-B-031), the Natural Science Foundation of China (Grant Nos. 61872113 and 62006061), and the Shenzhen Foundational Research Funding (JCYJ20200109113441941).

## Conflict of Interest

The authors declare that the research was conducted in the absence of any commercial or financial relationships that could be construed as a potential conflict of interest.

## Publisher’s Note

All claims expressed in this article are solely those of the authors and do not necessarily represent those of their affiliated organizations, or those of the publisher, the editors and the reviewers. Any product that may be evaluated in this article, or claim that may be made by its manufacturer, is not guaranteed or endorsed by the publisher.

## Abbreviations

ML, machine learning; TSS, transsphenoidal surgery; CD, Cushing’s disease; ROC, receiver operating characteristic curve; AUC, area under the curve; UFC, urine free cortisol; LDDST, low-dose dexamethasone suppression test; HDDST, high-dose dexamethasone suppression test; MRI, magnetic resonance imaging; BIPSS, bilateral inferior petrosal sinus sampling; IOMRI, invasion of cavernous sinus from MRI; LR, logistic regression; RF, random forest; MLP, Multiparametric Linear Programming; SVM, support vector machine.
